# Supplementary material for: Neural markers of category-based selective working memory in aging
Source: Neuroimage. 2019 Jul 1;194:163–73. doi: 10.1016/j.neuroimage.2019.03.033 (PMC6547047; doi:10.1016/j.neuroimage.2019.03.033)
Supplement: FH_manuscript_final_appendix [file mmc1.docx]

**Appendix**


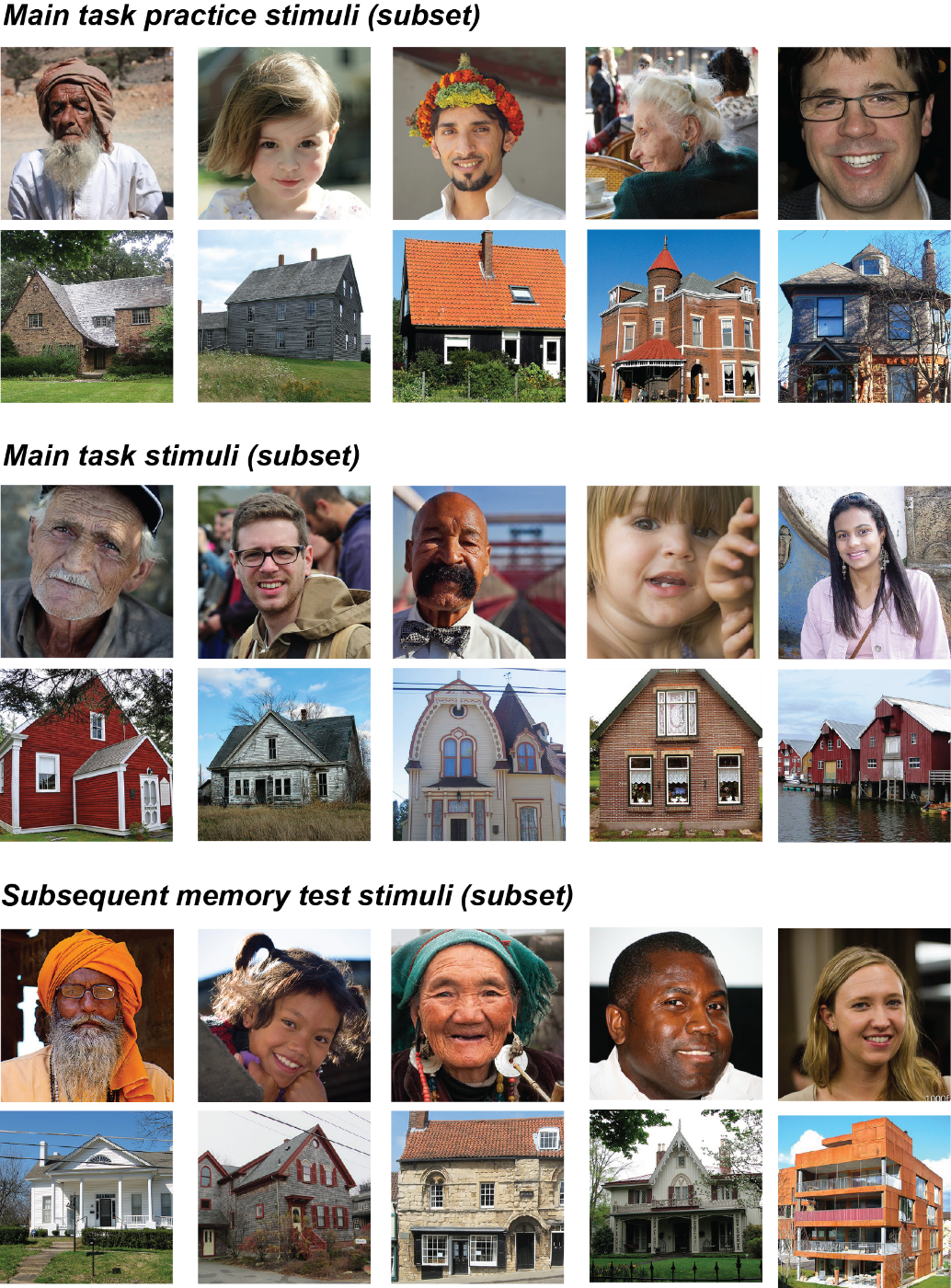


*Figure S1. Examples of stimuli used in the tasks. Top: Subset of the stimuli used in the practice session of main experiment (selective WM task). Middle: Subset of the stimuli used in the main experiment. Subset of the stimuli (‘new’ images not presented in the main experiment) used in the subsequent memory test.*

**fMRI cluster tables**

Naming guided by the Harvard-Oxford cortical and subcortical structural atlases

Abbreviations

fO: frontal operculum

IFG: inferior frontal gyrus

ITG: inferior temporal gyrus

LO: lateral occipital cortex

MTG: middle temporal gyrus

OFG: occipital fusiform gyrus

SPL; superior parietal lobule

| Location | Cluster number | Z value (local maxima) | x | y | z |
| --- | --- | --- | --- | --- | --- |
| Right ITG | 1 (cluster extent: 1066 voxels, p =0.001) | 3.83 | 50 | -54 | -12 |
| Right inferior LO | 1 | 3.25 | 50 | -68 | -10 |
| Right ITG | 1 | 3.18 | 44 | -36 | -14 |
| Right ITG | 1 | 3.13 | 58 | -38 | -14 |
| Right inferior LO | 1 | 2.99 | 46 | -78 | 2 |
| Right inferior LO, MTG | 1 | 2.92 | 44 | -60 | 8 |

*Table S1. Peak anatomical locations, Z values, and MNI coordinates from the significant cluster which exhibited a significant correlation between attentional modulation in the right LO and IT cortex for faces (FA > FI) and the subsequent memory difference score for face images (within brain regions that showed significant attentional modulation for FA > FI; with activation mask from the FA > FI contrast; see Methods).*

| Location | Cluster number | Z value (local maxima) | x | y | z |
| --- | --- | --- | --- | --- | --- |
| Left temporal occipital fusiform cortex, OFG | 1 (cluster extent: 2144 voxels, p =0.0003) | 3.91 | -22 | -60 | -10 |
| Left posterior temporal fusiform cortex, temporal OFG | 1 | 3.85 | -32 | -42 | -22 |
| Left temporal occipital fusiform cortex, posterior temporal fusiform cortex | 1 | 3.82 | -32 | -46 | -24 |
| Left temporal occipital fusiform cortex | 1 | 3.56 | -36 | -60 | -16 |
| Left ITG, posterior temporal fusiform cortex | 1 | 3.56 | -44 | -50 | -8 |
| Left temporal occipital fusiform cortex, OFG | 1 | 3.53 | -38 | -64 | -14 |
| Right temporal occipital fusiform cortex | 2 (cluster extent: 1044 voxels, p = 0.01) | 3.71 | 34 | -48 | -24 |
| Right temporal occipital fusiform cortex | 2 | 3.66 | 34 | -62 | -24 |
| Right temporal occipital fusiform cortex | 2 | 3.61 | 30 | -58 | -24 |
| Right temporal occipital fusiform cortex | 2 | 3.58 | 30 | -60 | -20 |
| Right OFG | 2 | 3.39 | 26 | -70 | -24 |
| Right inferior LO | 2 | 3.16 | 36 | -78 | -4 |

*Table S2. Peak anatomical locations, Z values, and MNI coordinates from the significant clusters which exhibited a significant correlation between attentional modulation in the PHG and IT cortex (HA > HI) and digit span (within brain regions that showed significant attentional modulation for HA > HI; with activation mask from the HA > HI contrast; see Methods).*

|  | Cluster number | Z value (local maxima) | x | y | z |
| --- | --- | --- | --- | --- | --- |
| Precuneus | 1 (cluster extent: 1318, p = 0.004) | 3.94 | -10 | -74 | 30 |
| Precuneus | 1 | 3.36 | -36 | -42 | 58 |
| Left SPL | 1 | 3.32 | -32 | -54 | 54 |
| Left cuneal cortex | 1 | 3.09 | -4 | -82 | 28 |
|  | 1 | 3.08 | -18 | -86 | 24 |
|  | 1 | 2.97 | -22 | -56 | 44 |
| Precuneus | 2 | 3.75 | 10 | -50 | 56 |
| Postcentral gyrus, precuneus | 2 | 3.45 | 10 | -38 | 58 |
| Postcentral gyrus | 2 (cluster extent: 993, p = 0.02) | 3.32 | 14 | -38 | 54 |
| Right superior LO, precuneus | 2 | 3.02 | 22 | -66 | 38 |
| Precuneus | 2 | 2.9 | 0 | -54 | 60 |
| Right precentral gyrus, | 2 | 2.85 | 8 | -22 | 62 |

*Table S3. Peak anatomical locations, Z values, and MNI coordinates from the whole-brain significant clusters showing areas that were functionally coupled with bilateral FG for ignoring face stimuli relative to encoding face stimuli (FI > FA).*

| Cluster Index | Cluster Index | Z | x | y | z |
| --- | --- | --- | --- | --- | --- |
| Right orbital frontal cortex | 2 (cluster extent: 4194, p = 1.19e-7) | 4.1 | 26 | 18 | -18 |
| Right ITG, temporal occipital fusiform cortex | 2 | 3.99 | 46 | -56 | -14 |
| Right inferior LO | 2 | 3.76 | 48 | -76 | -2 |
| Right inferior LO, ITG | 2 | 3.57 | 52 | -60 | -8 |
| Right Heschl's gyrus/transverse temporal gyrus | 2 | 3.48 | 56 | -8 | 2 |
| Right ITG, MTG | 2 | 3.46 | 54 | -54 | -10 |
| Right frontal pole | 1 (cluster extent: 821, p = 0.0475) | 3.39 | 42 | 36 | -18 |
| Medial frontal cortex, frontal pole | 1 | 3.32 | -2 | 54 | -10 |
| Right frontal pole | 1 | 3.19 | 38 | 38 | -14 |
| Ventral medial frontal cortex | 1 | 3.1 | -2 | 40 | -24 |
| Right medial frontal cortex | 1 | 3.07 | 10 | 50 | -10 |
| Right medial frontal cortex | 1 | 3.04 | 6 | 46 | -16 |

*Table S4.* *Peak anatomical locations, Z values, and MNI coordinates from the whole-brain significant clusters showing areas that exhibited a positive correlation (across subjects) between the degree of coupling with bilateral FG during selective WM for faces (FA > FI, PPI effect) and the subsequent memory different for faces.*

| Location | Cluster number | Z-value (peak) | x | y | z |
| --- | --- | --- | --- | --- | --- |
| Left fO, insular cortex | 1 (cluster extent: 3746, p = 5.36e-7) | 4.11 | -34 | 22 | 2 |
| Left frontal orbital cortex, insular cortex, fO | 1 | 3.83 | -38 | 22 | -4 |
| Left central opercular cortex | 1 | 3.6 | -50 | -22 | 18 |
| Left precentral gyrus | 1 | 3.52 | -36 | 0 | 26 |
| Left IFG pars opercularis | 1 | 3.34 | -44 | 10 | 22 |
| Left IFG pars opercularis | 1 | 3.32 | -48 | 10 | 24 |

*Table S5. Peak anatomical locations, Z values, and MNI coordinates from the whole-brain significant cluster showing areas that were functionally coupled with bilateral PHG during selective WM for house stimuli (HA > HI).*
